# High-resolution traffic flow data from the urban traffic control system in Glasgow

**DOI:** 10.1038/s41597-025-04494-y

**Published:** 2025-02-12

**Authors:** Yue Li, Qunshan Zhao, Mingshu Wang

**Affiliations:** 1 Urban Big Data Centre, School of Social and Political Sciences, University of Glasgow, Glasgow, UK; 2 School of Geographical and Earth Sciences, University of Glasgow, Glasgow, UK

**Keywords:** Geography, Interdisciplinary studies

## Abstract

Traffic flow data has been used in various disciplines, including geography, transportation, urban planning, and public health. However, existing datasets often have limitations such as low spatiotemporal resolution and inconsistent quality due to data collection methods and the need for an adequate data cleaning process. This paper introduces a long-term traffic flow dataset at an intra-city scale with high spatio-temporal granularity. The dataset covers the Glasgow City Council area for four consecutive years spanning the COVID-19 pandemic, from October 2019 to September 2023, providing comprehensive temporal and spatial coverage. Such detailed information facilitates diverse applications, including traffic dynamic analysis, traffic management, infrastructure planning, and urban environment improvement. Also, it provides a valuable dataset to understand traffic flow change during a once-in-a-lifetime pandemic event.

## Background & Summary

In most cities, motor vehicles dominate the transportation system^1^, which causes a series of challenges, such as traffic congestion, air pollution, and road accidents. Those traffic-related issues cause significant challenges in urban residents’ daily lives, and the solutions are still unclear^2–4^. To address these issues, the first step is to capture and understand the traffic flow information of motor vehicles in urban areas. To obtain such information, local governments have implemented Intelligent Transportation Systems (ITSs), incorporating diverse urban sensing technologies, including induction loop coil sensors on the road surface, above ground thermal and CCTV cameras. This system generates traffic flow data, enabling monitoring urban traffic flow patterns in high spatiotemporal resolution.

Traffic flow data has been widely applied in human geography, transportation engineering and management, urban planning, and public health^5^. It reflects the carriageway occupancy^6,7^ and records the number of motor vehicles at the road network level, indicating traffic congestion and underlying socioeconomic environments^5^. In transportation applications, traffic flow data has been used to study traffic surveillance, management, and prediction. In traffic surveillance, such data helps recognise traffic hotspots, identify traffic congestion, and monitor accident-prone areas^8^. Based on the surveillance, the local government manages the traffic by optimising traffic signal timings, providing alternative routing suggestions, and implementing safety measures. Moreover, future traffic conditions can be predicted by analysing historical traffic flow data and real-time traffic management.

As pointed out by several studies^9–11^, traffic flow data plays a key role and serves as a data foundation in analysing travel behaviours and patterns. Although some studies have released a set of traffic flow-related open datasets^7,12^ for urban traffic analysis, these datasets lack three major aspects. First, long-term traffic flow data is lacking at high temporal granularity. For instance, some datasets only provide traffic flow data at an annual level^13,14^, while some studies generate datasets in 5-minute intervals but only for one month^12^. Second, satisfactory spatial coverage at a city scale is not available. Most openly available datasets provide traffic flow information around the city centre or motorways, which always shows high traffic volume^7^. Higher spatial coverage datasets provide more detailed traffic patterns over the entire city, which are necessary to more accurately characterise heterogeneous relationships between mobility patterns of vehicles and surrounding socioeconomic environments within cities. Third, the quality and accuracy of data generated by ITSs are not controllable, especially for long-term traffic flow data. Various factors, including sensor precision, environment conditions, and algorithm complexities, can lead to unpredictable inaccuracies in the data.

To address the limitations of existing traffic flow datasets, we introduce an openly available dataset that provides city-scale traffic flow data within Glasgow City for four consecutive years from October 2019 to September 2023. This paper proposes a workflow to improve the quality of datasets via spatial and temporal data cleaning and processing. The traffic flow data has been collected via a network of sensors placed on street furniture or under the road surface itself^6^. The sensors are placed along roads ranging from motorways to local roads, encompassing all categories from main roads to fifth-class roads in the UK, and they record the traffic flow every 15 minutes. The dataset developed in this paper holds great potential for various research directions and downstream applications. Here are some potential benefits and applications:

- Traffic dynamic analysis at the intra-city scale. Researchers can conduct in-depth analyses of traffic patterns within the city, identifying (off) peak-hour trends, seasonal variations, and annual patterns.
- Traffic management and infrastructure planning. By analysing the traffic flows and their surrounding built environment, city planners and transportation agencies can optimise traffic signal timings, enhance road infrastructure, and manage congestion.
- Urban environment improvement. This four-year dataset can aid in assessing the environmental impact of traffic flows, helping researchers understand the relationship between transportation patterns and air quality and improving the urban environment.

## Methods

**Fig. 1:**
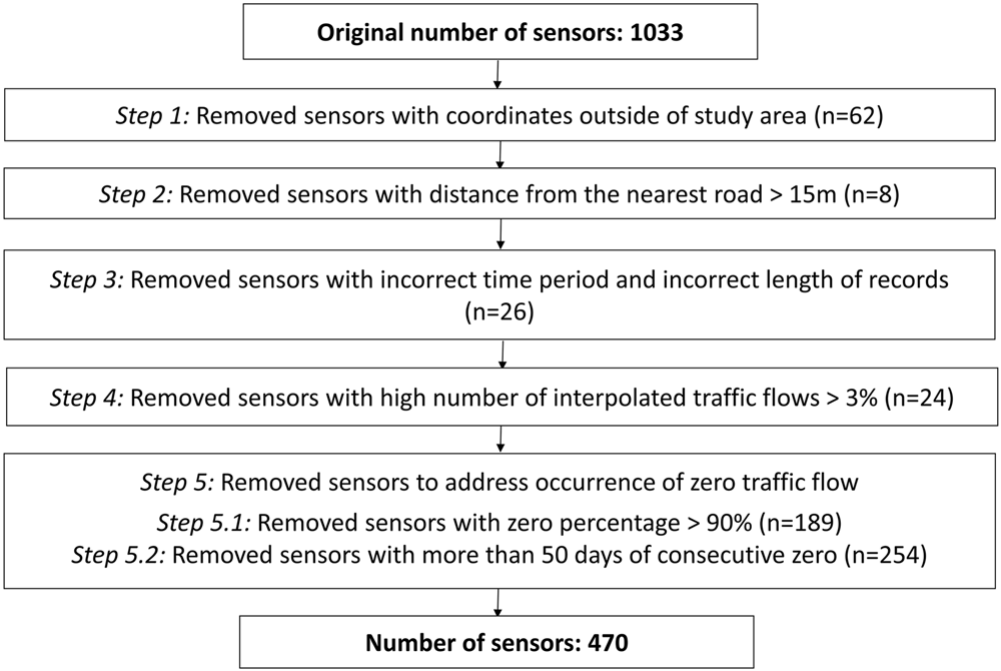
Data cleaning flowchart.

Figure 1 illustrates the detailed process involved in generating the traffic flow dataset. Initially, we collect the raw traffic flow data through the Glasgow open data portal^15^ (https://gcc.developer.azure-api.net/api-details#api=traffic&operation=5b044adda611ad4c9b1c58b2), which provides several open data Application Programming Interfaces (APIs) by the Glasgow City Council. This dataset is derived from a network of over 1000 sensors positioned along roads, capturing traffic information at 15-minute intervals. To refine the dataset, we implemented a two-fold filtration process based on spatial and temporal constraints. Initially, data is filtered according to the sensors placed, narrowing the scope to specific geographical areas and locations. Subsequently, a temporal constraint is applied to refine the dataset based on the study period and 15-minute intervals. Then, we examined the specific information in the records for the remaining sensors. In this paper, each record refers to the traffic flows captured by the sensors at one-time intervals. Sensors with a substantial proportion of irregular traffic flows or incorrect time intervals are excluded from the dataset. The final step involves reconstructing and aggregating the cleaned traffic flow data. This process ensures that the data adheres to the specific time intervals and formatting requirements, resulting in an accurate and reliable dataset for downstream applications.

### Original data Sources

**Fig. 2:**
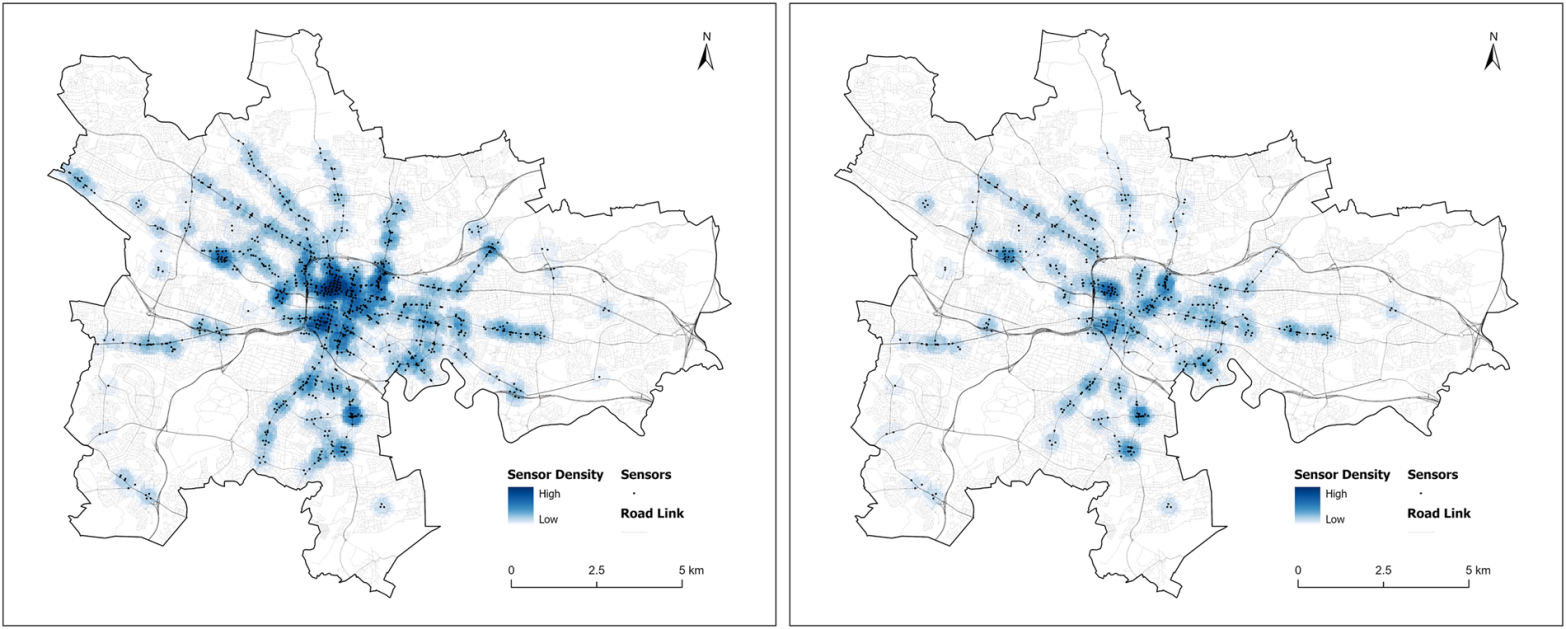
Spatial distribution of original(left) and filtered(right) traffic sensors in Glasgow.

The original traffic flow data in Glasgow is provided by Glasgow City Council through its open data portal^15^ (https://gcc.developer.azure-api.net/api-details#api=traffic&operation=5b044adda611ad4c9b1c58b2). Glasgow City Council manages infrastructure to monitor and control real-time traffic flow data across the city streets. The data is collected via a Split Cycle Offset Optimisation Technique (SCOOT) based Urban Traffic Control (UTC) system^6^. The most common sensors SCOOT uses are ones buried in the road surface, such as an inductive loop or mounted above ground, usually on top of the signal post, such as microwave vehicle detectors. Inductive loops collect traffic flows by analysing the electromagnetic effects caused by the presence or passage of a vehicle^16^. Specifically, when a vehicle passes the inductive loop, SCOOT converts the information into a “link profile unit” (lpu), which is a hybrid of traffic flow and occupancy. The unit used by SCOOT in its calculations is called “Cyclic flow profiles”, which is the lpu signals over time that are constructed for each link. This paper has a total of 1033 sensors collecting the traffic flows for four years from October 2019 to September 2023. The detailed spatial density distribution of the physical sensor placement is shown in Fig. 2. The sensors are located from the main roads (motorways) to the fifth-class roads (local roads) in Glasgow, recording the traffic flows at 15-minute intervals. This extensive sensor network provides widespread coverage of the city’s diverse roads, contributing to an in-depth analysis of traffic patterns and surrounding built environments.

### Data refinement

Since the raw traffic flow data is collected via the automatic traffic control system sensors, inspecting and cleaning the dataset before proceeding with any downstream analysis is necessary. We first filter the data according to the sensors placed with spatial and temporal constraints.

*Step 1*. The coordinates of each sensor are extracted, and a comparison is made with the geographic boundaries defining the Glasgow City Council area. Sensors located outside this specific area are deemed beyond the scope of the research and consequently excluded from further steps.

*Step 2*. We consider the spatial relationship between traffic flows and the road network. This process evaluates the proximity of the sensors to the city’s road infrastructure. Since sensors record traffic flows in Glasgow along the road network, it is essential to consider the range of Euclidean distances from each sensor to its nearest road. In Glasgow, over 99% of sensors are placed within a 15-meter radius of roads. Only eight sensors deviate by 20 meters or more, resulting in their exclusion from the dataset.

*Step 3*. We implement temporal filtering on the traffic flow data. The dataset in this paper spans from October 1st, 2019, to September 30th, 2023, including a total of 1461 days. Sensors with traffic flows starting later than October 2019 or ending before September 2023 are excluded from the dataset. With a 15-minute collection interval, each sensor theoretically generates 140246 records. Five sensors that lacked valid data for over half the period were removed from this dataset. 99.4% of sensors operate typically, showing a loss of less than 5% of traffic flows over the four-year duration. Overall, 26 sensors are excluded based on the temporal constraint.

*Step 4*. After completing the spatial and temporal inspections detailed above, the traffic flow data undergoes numerical filtering. Glasgow City Council has implemented an interpolation method for instances without data returns. This method duplicates the number of flows from the latest to the current timestamp to address the absence of valid data. Subsequently, 24 sensors in Glasgow were identified and removed due to having more than 15% interpolated data, while the remaining sensors exhibit interpolation levels below 3%.

*Step 5*. The most prevalent data issue in the time-series dataset is the occurrence of zero traffic flow values. These zeros indicate extended periods when no vehicles pass a particular road, lasting for hours or even days. We address this issue by analysing zero traffic flow from two different aspects.

*Step 5.1*. First, we assess the frequency of no-data records for each sensor. We calculate each sensor’s overall percentage of zero traffic flow values across all four-year records. We exclude 189 sensors that showed a high frequency of no-data, with more than 90% zero traffic flow during the four years.

*Step 5.2*. Next, we focus on long and continuous periods of no-data events. For each sensor, we identify all the entire natural days with 24 hours of zero traffic flow. This step assumes that at least one vehicle should pass each sensor daily in Glasgow. The number of no-data natural days for each sensor varies across sensors, ranging from a maximum of 1164 to a minimum of 50 days. To ensure data integrity and completeness, we retain only sensors with 50 days of no-data events. After this refinement, a total of 470 sensors are included in the dataset, with each sensor recording 1,411 days of data out of 1,461 days across four years.

### Data aggregation

In this paper, we reconstruct and aggregate the raw traffic flows hourly. Specifically, we eliminate the interpolated records for each retained sensor, replacing them with NaN (Not a Number) traffic flow values. The hourly traffic flows are aggregated by first computing the average value for each hour. This computation ignores NaN values, and the flows are averaged based on the valid number of records. For instance, if two valid flows are available for 8 AM on August 16th, 2021, the sum of the two records is divided by two. Then, we multiplied the averaged flows by four, as each hour should ideally have four records with a 15-minute interval. An hour with four invalid records is removed from the data, as the aggregation result is considered invalid. We will not include any invalid NaN values in the final datasets.

**Fig. 3:**
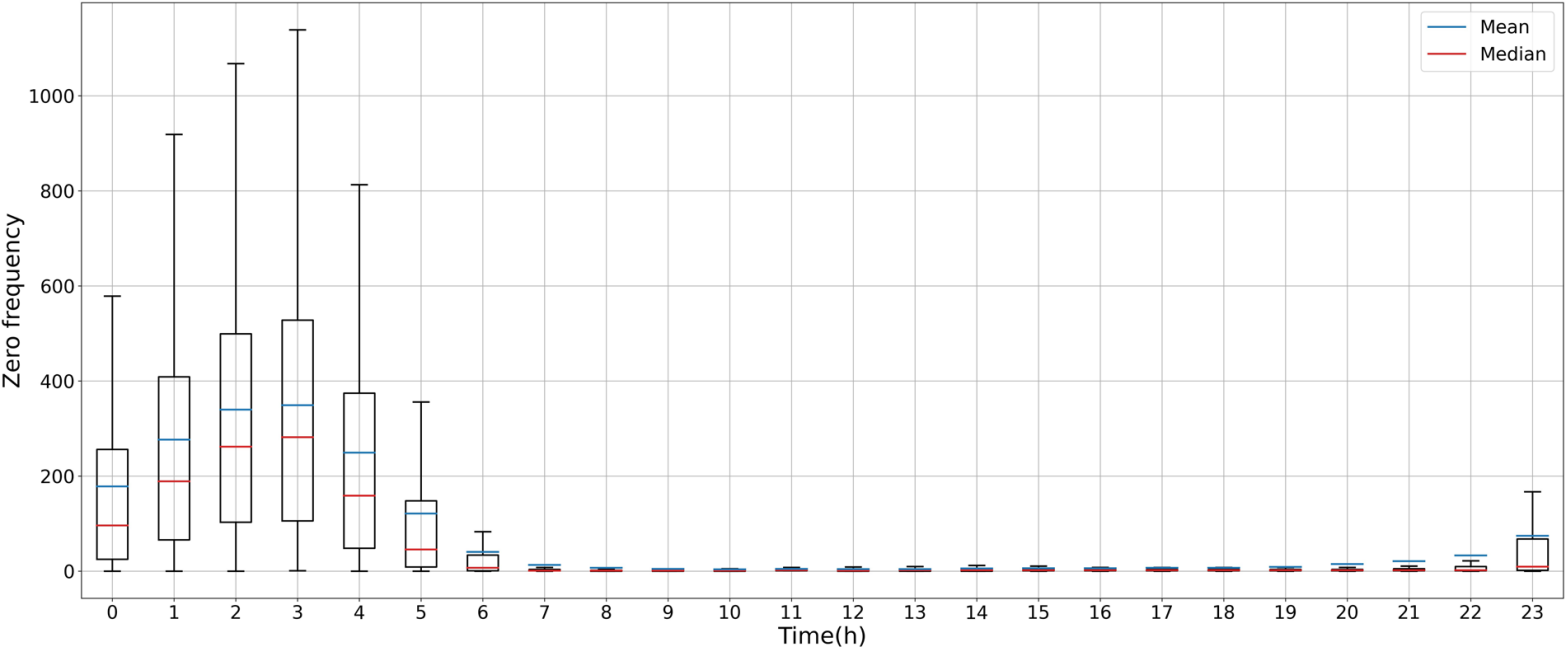
Boxplot of hourly zero flow frequency.

**Fig. 4:**
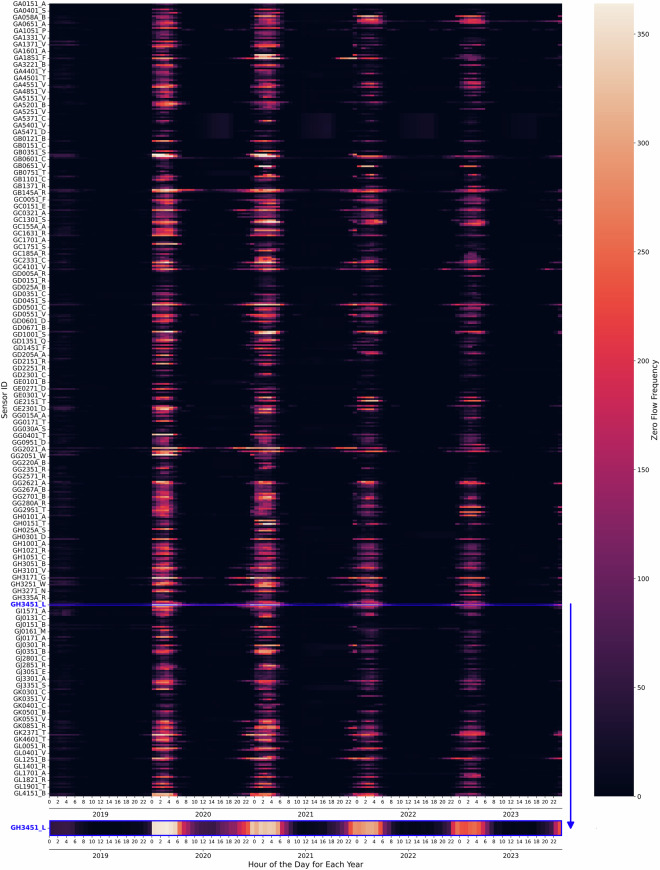
Heatmap of hourly zero flow frequency.

In particular, the zero values of the retained 470 sensors are kept, as their occurrence is considered reasonable, as illustrated in Fig. 3. Figure 3 is a box plot illustrating the zero flow frequency for each sensor over four years, segmented by each hour of the day. Figure 4 is a heat map showing the frequency of zero flow events, where the x-axis represents the hours of the day over four consecutive years, and the y-axis corresponds to each sensor. Since traffic flow data collection began in October 2019, the zero flow frequency is relatively low in 2019 due to the limited data volume. Figure 4 shows that the zero flow frequency was higher in 2020 and 2021 compared to 2022 and 2023 due to the mobility restrictions during the COVID-19 pandemic. This pattern supports the validity of the observed zero flow events in the remaining dataset. Both the line plot and the heat map demonstrate that, for most sensors, zero traffic flows were recorded before 6 AM and after 11 PM. Specifically, 3 AM was the quietest time in Glasgow. Sensor GH3451_L (highlighted in Fig. 4), located at the car park entrance of Emirates Arena, exhibited the highest zero flow frequency at 3 AM for four consecutive years. This indicates that no vehicles came to Emirates Arena at 3 AM for more than 88% of the recorded days. The busiest time in Glasgow was around noon, with over 99% of days showing traffic flows during this period.

## Data Records

The dataset is available from a long-term Zenodo repository^17^. The records of this dataset are compiled using three main products. The first contains 470 files, each containing a sensor’s hourly traffic flow data from October 1st, 2019, to September 30th, 2023. The second product is a single file providing geographical information for 470 sensors. The third product is a single file that includes binary results for each data cleaning step applied to the original 1033 sensors, including their geographical information as well. All files are formatted in comma-separated values (CSV), a widely employed format for publicly storing, transferring, and sharing data.

### Original sensor status

This file provides information on the status of sensors through multiple data cleaning steps, including their unique ID, geographical coordinates (latitude and longitude), and binary results for each step (steps 1 to 5.2). Each row in the file represents a sensor, specifying whether it was remained or removed at current step of the analysis. The removed sensors are marked with a value of 0, while the remaining sensors are marked with 1. This structured file helps researchers understand the filtering process and identify sensor issues across different steps. The attributes of the file are presented in Table 1.

**Table 1:** Description of sensor status attributes.

File name	Column name	Description
status.csv	id	Unique ID for each sensor, e.g., GA0601_T.
	latitude	The Latitude in decimal degrees of WGS84 coordinates, e.g., 55.86238129.
	longitude	The Longitude in decimal degrees of WGS84 coordinates, e.g., −4.26570708.
	step 1	The status of sensors at the current step is 1 if retained and 0 if removed.
	step 2	
	step 3	
	step 4	
	step 5.1	
	step 5.2	

### Sensor locations

This file provides sensor information, including ID and the corresponding geographical coordinates. Each row in the file includes details about a sensor, specifying the unique ID with the Longitude and Latitude coordinates in the WGS84 projection system. By mapping the spatial distribution of sensors across Glasgow, researchers can gain insights into various studies, such as analysing spatial patterns, identifying hotpots, and understanding the impact of geographical features on built environments. Table 2 shows the attributes of sensor locations.

**Table 2:** Description of sensor location attributes.

File name	Column name	Description
locations.csv	id	Unique ID for each sensor, e.g., GA0601_T.
	latitude	The Latitude in decimal degrees of WGS84 coordinates, e.g., 55.86238129.
	longitude	The Longitude in decimal degrees of WGS84 coordinates, e.g., −4.26570708.

### Traffic flows

Traffic flow data consists of 470 files, each corresponding to a specific sensor and covering four years. Table 3 describes the files, while Table 4 provides data attributes. Each row in the files contains information about the specific date and time the traffic flow data was collected. There are 50 missing days in this dataset for four years from October 1st, 2019, to September 30th, 2023. Table 5 details the missing days for all the sensors in the traffic flow data.

**Table 3:** Data files of traffic flows.

File name	Description
[sensor_id].csv	Traffic flows. [sensor_id] refers to the ‘id’ from the locations.csv

**Table 4:** Description of traffic flow data attributes.

Column name	Description
date	The date the data is collected (YYYY-MM-DD), e.g., 2021-11-04.
time	The hours of the day the data is collected range from 0 to 23, 0 = [0,1), 23 = [23,24).
flow	Number of vehicles that pass the sensor location during the one-hour interval.

**Table 5:** Missing dates of data from all sensors.

Year	2019	2022	2023
Date	10/17/2019 to 11/19/2019	04/09/2022, 04/10/2022	04/23/2023, 07/13/2023 to 07/25/2023

## Technical Validation

This section aims to validate our dataset’s quality and technical reliability through various methods. First, we analysed the spatial distribution of original and filtered sensors. Second, we analysed the temporal distribution of traffic flows during the whole study period. Third, we compared the daily average traffic flow with the stringency index across different stages of the COVID-19 pandemic, providing insights into the consistency of variation between traffic flows and external factors influencing traffic behaviours. To further ensure data reliability and integrity, we examined daily traffic flow patterns and the frequency of zero values in our traffic flow dataset. These evaluations comprehensively understand the reliability and usefulness of traffic flow datasets.

### Spatial distribution of sensors

In this study, we apply the chi-square test to evaluate the original and filtered sensors distribution in different land cover and road type categories. The chi-square test is a statistical method used to determine whether there is a statistically significant difference between the expected and observed frequencies in one or more categories of a contingency table. For each categorical variable, land cover and road types, we constructed a contingency table (Table 6) that records the frequency of each category within both groups. The chi-square test statistic is calculated using the formula^18^:

$${\chi }^{2}=\sum \frac{{(O-E)}^{2}}{E}$$

Where O represents the observed frequency of each category, which is the number of sensors in each land cover and road type for filtered sensors. E is the expected number of sensors in each land cover and road type derived from original sensors. The ${\chi }^{2}$ statistic is used to test whether there is a statistically significant spatial distribution difference between two groups of sensors at different categories of land cover and road types. The p-value of the chi-square test is 0.94794, which is higher than 0.05 and very close to 1. This high p-value indicates no statistically significant difference between the distributions of the original and filtered sensors at different land cover and road types, suggesting the spatial distributions of filtered sensors are similar to those of original sensors. In this case, we can confirm that filtered sensors are a good representative sample of the original sensors.

**Table 6:** Frequency of road type and land use category for orginal and filtered sensors.

Data	Category	Original Sensors	Filtered Sensors
Land Use^33^	Construction Sites	1	1
	Continuous Urban Fabric (S.L.: >80%)	34	18
	Discontinuous Dense Urban Fabric (S.L.: 50%–80%)	66	41
	Discontinuous Medium Density Urban Fabric (S.L.: 30%–50%)	2	1
	Fast Transit Roads and Associated Land	21	6
	Forests	2	0
	Green Urban Areas	20	10
	Industrial, Commercial, Public, Military and Private Units	106	54
	Other Roads and Associated Land	707	336
	Railways and Associated Land	7	3
	Sports and Leisure Facilities	3	0
	Water	2	0
Road Types^36^	A Road	346	168
	A Road Primary	52	28
	B Road	85	47
	Local Access Road	3	1
	Local Road	122	53
	Minor Road	338	163
	Motorway	17	5
	Restricted Local Access Road	6	5
	Restricted Secondary Access Road	2	0

### Temporal distribution of daily traffic flows

**Fig. 5:**
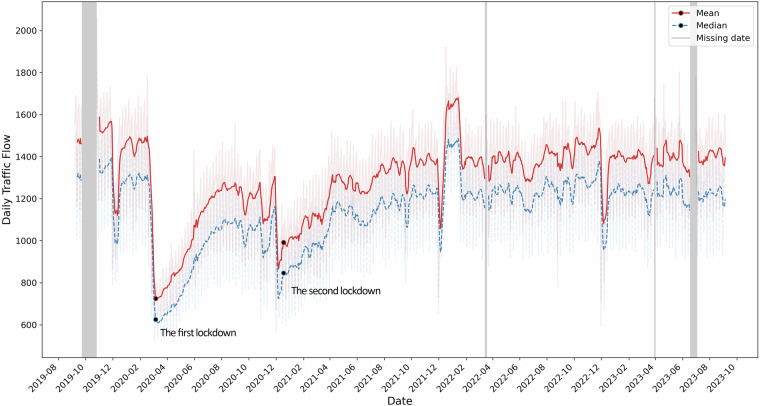
Daily traffic flows from October 1st, 2019, to September 30th, 2023.

Figure 5 shows the mean and median value of daily traffic flows over 470 sensors in Glasgow, with missing dates (Table 5) highlighted in grey. During the four years, the daily traffic flows consistently decreased dramatically in late December due to Christmas and rebounded to the level of average days at the beginning of January. However, the outbreak of the COVID-19 pandemic and social distancing measures restricted human mobility in the UK^19,20^. Following the first country-wide lockdown imposed on March 23^rd^, 2020^21^, there was a sharp decline in daily traffic flows across Glasgow. There was a gradual increase in traffic flows with the easing of restrictions, but it did not reach pre-lockdown levels. In January 2021, the UK government introduced a second lockdown, similar to it in March 2020^21^. This lockdown started following the Christmas period, slowing down the anticipated traffic recovery from holidays in Glasgow. The annual fluctuations of Christmas and the responses to emergency and government policies demonstrate the reliability and consistency of our traffic flow dataset.

### Comparison with Stringency Index

The Stringency Index is a metric used to quantify the restrictions on mobility in response to the COVID-19 pandemic. The restrictions on mobility are evaluated by the Oxford COVID-19 Government Response Tracker (OxCGRT)^22^. The OxCGRT records the government responses through 21 indicators representing different policy measures. Specifically, eight indicators measure containment and closure policies, such as school and workplace closures. Another eight indicators measure healthcare responses, including facial coverings and vaccination policies. The remaining four indicators reflect economic policies, such as income support and fiscal measures, with another miscellaneous indicator. The Stringency Index is calculated as an average of the eight containment and closure indicators and one healthcare indicator, quantifying the strictness of policies that primarily restrict people’s behaviour^20,21^. The Stringency Index ranges from 0 (no restrictions) to 100 (most stringent restrictions). To better compare traffic flow patterns with the introduction of restrictions, we convert the Stringency Index into the Freedom of Association Index^20^, which is calculated as follows:

$${\rm{Freedom\; of\; Association\; Index}}=100-{\rm{Stringency\; Index}}$$

**Fig. 6:**
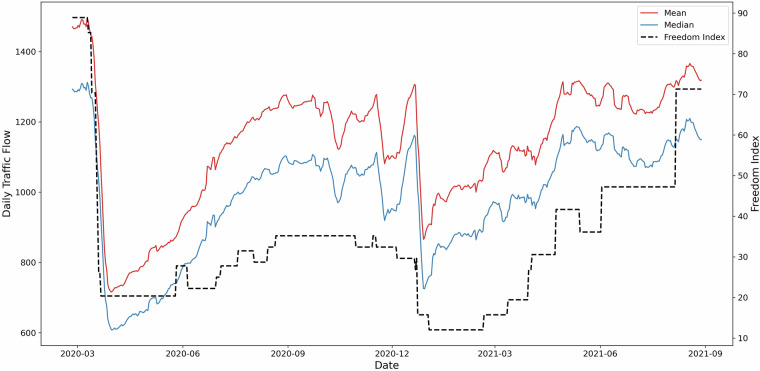
Daily traffic flows and freedom of association from March 1st, 2020, to August 31st, 2021.

The Freedom of Association Index reflects the degree of freedom individuals have to associate and gather socially, providing a complementary perspective to the Stringency Index. Figure 6 illustrates a positive relationship between daily traffic flows and the Freedom of Association Index. Both the mean and median traffic flows declined significantly, corresponding to reductions in the Freedom of Association Index observed in March 2020 and January 2021. Conversely, as the Freedom of Association Index increased, indicating fewer restrictions on social interactions, traffic flows demonstrated a corresponding rise. To further validate the traffic flow dataset, Pearson’s correlation coefficient^23–25^ and Spearman’s correlation coefficient^26,27^ were employed to assess the degree of correlation between the Freedom of Association Index and daily average traffic flows. The results (Table 7) indicate a high consistency and strong correlation between the two datasets, confirming the validity and reliability of the traffic flow dataset in Glasgow.

**Table 7:** Correlation coefficients between the Freedom of Association index and daily average traffic flows.

	Coefficient	P value
Pearson	0.751	5.134e-101
Spearman	0.861	2.326e-163

### Hourly traffic flows and zero frequency

**Fig. 7:**
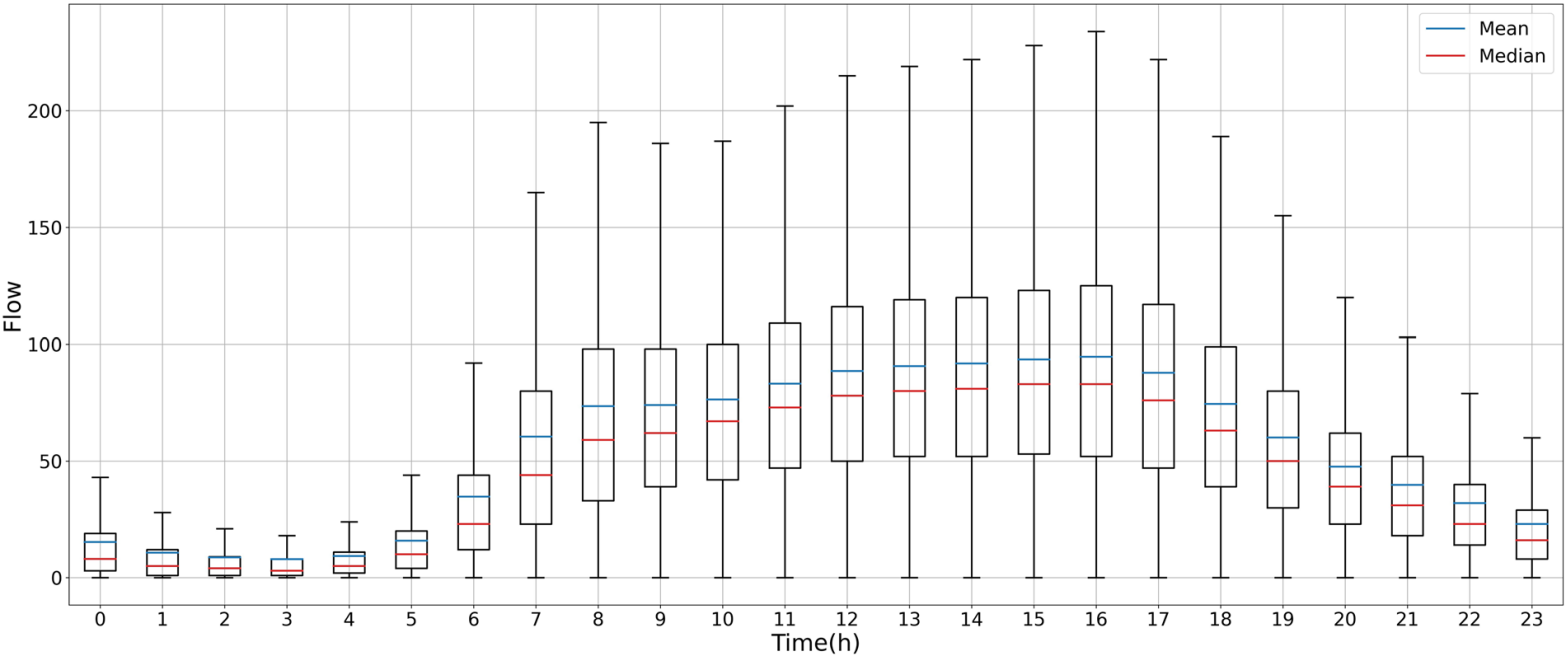
Boxplot of hourly traffic flows.

Figure 7 demonstrates the statistical distribution of hourly traffic flows in Glasgow over 24 hours through four consecutive years. The hourly data demonstrates a consistent pattern of increasing traffic flows from morning, starting as early as 6 AM. The traffic flows continued to rise steadily, indicating sustained high demand throughout the daytime until 4 PM. From 5 PM onwards, there was a significant decrease in hourly traffic flows, reaching its lowest point at 3 AM the following day. The hourly zero frequency shows the opposite trend in Fig. 3, where the highest frequency of zero traffic flow was observed at 3 AM. Conversely, the minimal instances of zero traffic flow were recorded from 7 AM to 10 PM. The correlation coefficient values in Table 8 further validate this relationship. Pearson and Spearman’s coefficients are lower than −0.8, indicating a strong negative correlation between hourly traffic flows and zero frequency. Therefore, our traffic flow data reliably captures the fluctuations in traffic throughout the day.

**Table 8:** Correlation coefficients between zero frequency and hourly average traffic flows.

	Coefficient	P value
Pearson	−0.803	2.306e-06
Spearman	−0.908	9.133e-10

### Morning peak comparison

**Fig. 8:**
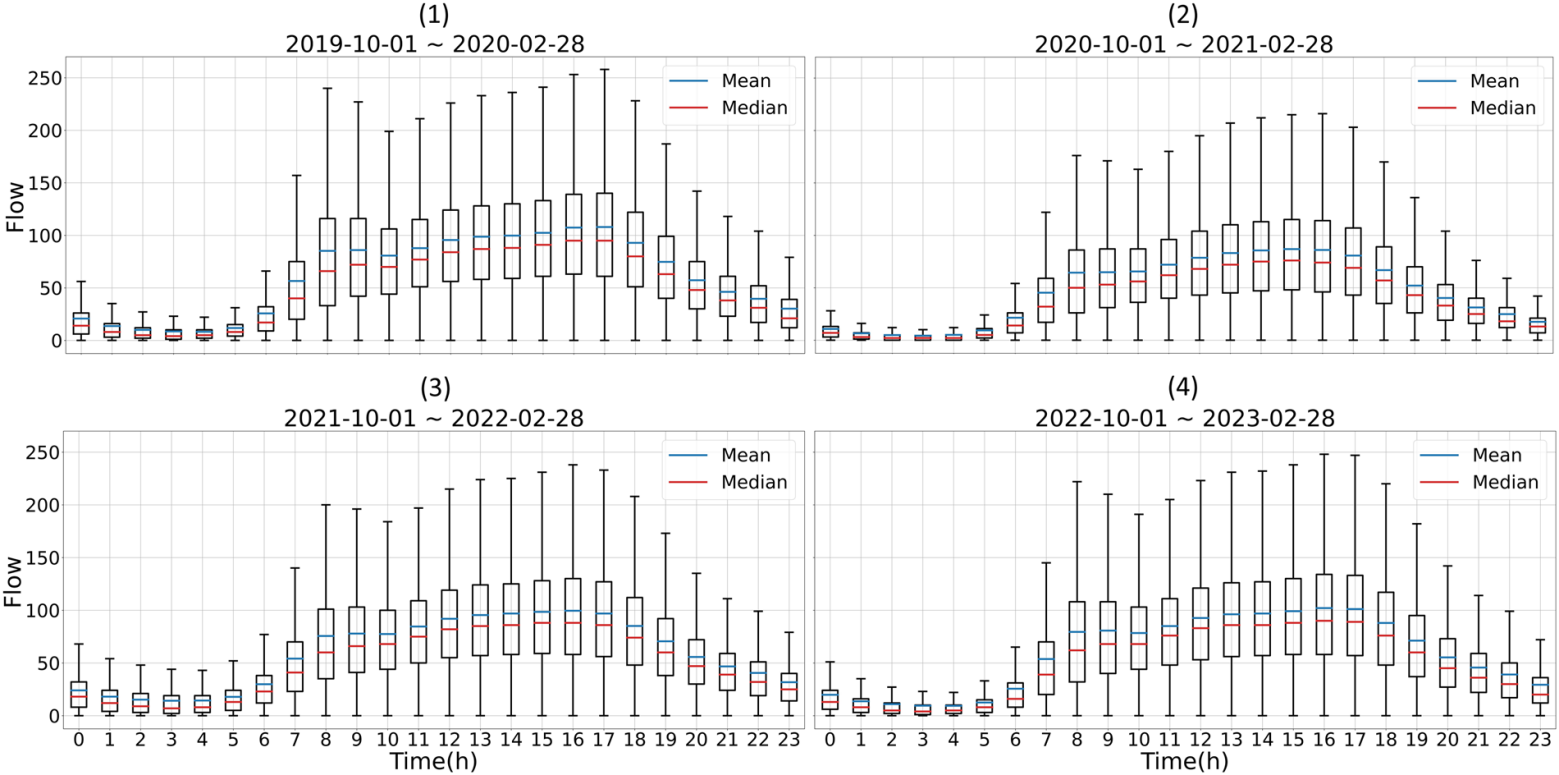
Boxplot of hourly traffic flows for four separate years.

Figure 8 shows hourly traffic flows for 24-hour periods in different years. We compare the mean and median traffic flows during the morning rush from 8 AM to 9 AM. It can be seen that there was a minor peak during the morning rush hours in Fig. 8 (1). However, the morning peak gradually disappeared in Fig. 8 (2)-(3). That might be because, during COVID-19, many people started working from home, reducing the need for commuting during traditional rush hours^28^. This trend highlights the significant impact of the pandemic on daily travel patterns and commuting behaviours^29^. In Fig. 8 (4), the morning peak began to reappear as restrictions eased and people returned to their workplaces. However, the post-pandemic morning peak did not return to its pre-pandemic levels. The pandemic has brought about lasting changes in work patterns, with many companies adopting hybrid or fully remote work models. This shift could lead to a long-term reduction in morning peak traffic, even after the pandemic.

## Usage Notes

As briefly mentioned in the ‘Background & Summary’ section, this dataset can be used in various aspects. Since it is long-term traffic flow data with hourly intervals, it can be used for traffic dynamic analysis across multiple time scales, including identifying short-term peak-hour trends, long-term seasonal variations, and annual patterns. Those features can be coupled to official weather observations^30^, which are recorded at the same temporal scale. By combining the hourly weather observations with traffic flows, users can make long/short-term traffic flow predictions. The temporal analysis can be done using various statistical software, such as R or Python programming languages or SPSS.

This intra-city scale traffic flow data can be coupled to the surrounding built environment for spatial analysis. Some examples are the road network data and Points of Interest available from the EDINA Digimap Service platform^31,32^ or land use data^33^. Moreover, this dataset can be applied to assess the environmental impact of traffic flows, such as the variation of air quality^34^ of established low-emission zones in Glasgow^35^. The spatial relationship can be analysed with Network Analyst or Buffer tools using conventional geographic information (GIS) software, such as ArcGIS, QGIS or packages for spatial data analysis for R or Python programming languages, such as rgeos, igraph, and GeoPandas.

## Data Availability

The code implementation was performed in Python using Jupyter Notebook. All the source codes for producing this dataset are available from the link: https://github.com/YueLi-0816/TrafficFlowData.
